# The use of home-based HIV testing and counseling in low-and-middle income countries: a scoping review

**DOI:** 10.1186/s12889-019-6471-4

**Published:** 2019-01-31

**Authors:** Moshoeu Prisca Moshoeu, Desmond Kuupiel, Nonjabulo Gwala, Tivani P. Mashamba-Thompson

**Affiliations:** Department of Public Health Medicine, School of Nursing and Public Health, University of KwaZulu-Natal, Durban, 4001 South Africa

**Keywords:** Home-based HIV testing and counselling, Use, Low-and-middle income countries

## Abstract

**Background:**

Knowledge of HIV status is crucial for both prevention and treatment of HIV infection. However, according to the Joint United Nations Programme on HIV/AIDS in low-and-middle-income countries (LMICs), only 10% of the population has access to HIV testing services. Home-based HIV testing and counseling (HTC) is one of the approaches which have been shown to be effective in improving access to HIV testing in LMICs. The objective of this review was to map evidence on the use of home-based HTC in LMICs.

**Methods:**

We searched PubMed, EBSCOhost, Google Scholar, Science Direct, World Health Organization library database and UNAIDS databases from January 2013 to October 2017. Eligibility criteria included articles pertaining to the use of home-based HTC in LMICs. Two reviewers independently reviewed the articles for eligibility. The following themes were extracted from the included studies: use, feasibility and effectiveness of home-based HTC on patient-centered outcomes in LMICs. The risk of bias for the included studies was assessed using mixed methods appraisal tool -version 2011.

**Results:**

A total of 855,117 articles were identified from all the databases searched. Of this, only 17 studies met the inclusion criteria after full article screening and were included for data extraction. All included studies presented evidence on the use of Home-based HTC by most age groups (18 months to 70 years) comprising of both males and females. The included studies were conducted in the following countries: Zambia, Uganda, South Africa, Kenya, Ethiopia, Malawi, Swaziland, Pakistan, and Botswana. This study demonstrated that home-based HTC was used in LMICs alongside supervised HTC intervention using different types of HTC tests kits produced by different manufacturers. This study also showed that home-based HTC was feasible, highly effective, and increased uptake of HIV testing and counseling. This study further demonstrated a highly successful usage of supervised home-based HTC by most age groups in LMICs**,** with majority of users being females (89.1%).

**Conclusion:**

We therefore recommend primary studies in other LMICs to determine the feasibility and use of HTC to help achieve the UNAIDS 90:90:90 targets. Interventions to improve the use of home-based HTC by males are also recommended.

**Trial registration:**

PROSPERO registration number: CRD42017056478.

**Electronic supplementary material:**

The online version of this article (10.1186/s12889-019-6471-4) contains supplementary material, which is available to authorized users.

## Background

The Joint United Nations Programme on HIV/AIDS (UNAIDS) 2017 global report on HIV indicated that there are over 36 million persons living with HIV, and millions of individuals have died as a results of HIV/AIDS-associated causes since the start of the epidemic [1, 2]. Although HIV new infections still occur worldwide, nearly two-thirds of all HIV new infections are recorded in Sub-Saharan Africa (SSA), particularly in Eastern and Southern Africa (approximately 42.5%) [3, 4]. Knowledge of one’s HIV status is extremely important for both the prevention and initiation of treatment [2, 5]. However, the UNAIDS estimates shows that, only 10% of the population have access to HIV testing services in low-and-middle income countries (LMICs) [6]. This poor access to HIV testing services is partly as a results of the stigma and discrimination associated with HIV, which makes people unwilling to voluntarily avail themselves for HIV testing [4].

Home-based HIV testing and counseling (HTC) is one of the approaches to HIV testing services and it has been demonstrated to be acceptable, feasible, and improving access to HIV testing [5, 7]. Home-based HTC, over-the-counter HIV testing was approved in July 2012 by the Food and Drug Administration in the United States of America [8]. A study conducted among men who have sex with men (MSM) in Australia found oral fluid-based HIV self-testing (HIVST) to be highly acceptable, using a supervised or an unsupervised approach [9]. Sabapathy et al., 2012 systematic review and meta-analysis of 21 observational studies in five African countries also reported that home-based HTC has the potential to significantly increase awareness of previously undiagnosed persons about their HIV status [10]. The World Health Organization (WHO) has also published guidelines for service providers and policy-makers on the delivery of home-based HTC [11, 12]. The home-based HTC provider’s primary responsibility includes meeting individuals in their homes and providing a client-centered pre and post-testing counseling sessions, conducting HIV testing, and linking clients to appropriate care and follow-up services [12, 13]. The costs of home-based HTC comparatively is said to be favorable to other HTC delivery strategies [14]. In generalized epidemics, home-based HTC represents a remarkable opportunity to test couples, children, and families in order to increase early identification of HIV-positive cases and to identify first-time testers [15]. HTC is the gateway to antiretroviral treatment and it is said to be associated with the adoption of protective behaviors, particularly among people living with HIV [12, 16]. Moreover, previous studies have indicated that home-based HTC greatly increases uptake of HTC and helps identify new HIV cases at earlier stages of the disease [17–19].

We have different types of HIV testing and counseling approaches such as facility based HIV testing, HIV self-testing (HIVST) and mobile HTC services. Facility-based HIV testing or client-initiated voluntary counselling and testing (VCT) involves individuals actively seeking HIV testing and counseling at a facility that offers these services [2, 20, 21]. HIVST is performed by a self-tester aided by a health care professional or without health professional [2, 8]: HIVST improves the frequency of testing among persons at highest risk of HIV infection, and facilitate mutual HIV testing with sex partners [2, 9]. However, home-based HTC provides an opportunity to reach many more people, removing logistical constraints and stigma often associated with facility-based HTC [17]. Home-based HTC additionally, may address the challenges of limited access to healthcare facilities where low access and limited reach of facility-based HIV testing services have been an impediment to global efforts to scale-up of HIV testing at the population level [22]. Moreover, the home-based HTC approach has received widespread international support. In view of this, the objective of this study was to map evidence on the use of home-based HTC in LMICs. The contribution of this scoping review is important and relevant through its capacity to demonstrate the current data in order to identify research gaps for future research and influence policy makers.

## Methods

### Study design

This study’s protocol is a part of a larger study with the title “the use and acceptability of Home-Based HTC in LMICs” registered in PROSPERO [23]. In this study, a scoping review was selected as the best method to map literature on the use of home-based HTC in LMICs. Scoping reviews are aimed at mapping the key concepts underpinning a research area and the main sources and types of evidence available and can be undertaken as stand-alone projects in their own right, especially where an area is complex or has not been reviewed comprehensively before [24]. The study was guided by the Arksey & O’Malley‘s framework [25]. The Arksey & O’Malley‘s framework stipulates the following steps: identification of the research question; identification of relevant studies; study selection; charting the data; and collating, summarizing, and reporting of the results [25–27]. We searched cross-sectional studies, randomized controlled trials, non-randomized controlled trials, observational studies, review articles, case reports and systematic reviews that examined the use and acceptability of home-based HTC in LMICs. We also included a quality assessment stage as proposed by Levac et al., 2010 [28]. The results of the review were presented according to the preferred reporting items for systematic review and Meta-analysis (PRISMA) guidelines [29].

### Literature search

We conducted a systematic literature search in the following databases to source the most relevant literature: PubMed, EBSCOhost (CINAHL and MEDLINE) Google Scholar, and Science Direct for articles pertain to the use of home-based HTC in LMICs. The search terms included: “Home-based HIV tests and counseling”, “use of home-based HIV testing and counseling”, as well as “acceptability of home-based HIV testing and counseling”. We utilized Boolean terms (AND/OR) to separate the key words as well as medical subject headings (MeSH) terms. Language of publications and type of study design limitations were removed. However, year of publication was limited to between January 2013 and October 2017 in order to obtain the most recent literature on home-based HIV testing and counseling in LMICs. We also searched grey literature such as reports on World Health Organization and UNAIDS websites; unpublished manuscripts; and theses and dissertations reporting evidence on home-based HTC in LMICs. Reference lists of the included studies were also searched to source relevant literature.

### Eligibility for research question

The research question for our study was: What is known from the existing literature about the use of home-based HIV testing in low and middle-income countries? An amended PICO (Population, Intervention, Comparison, and Outcomes) framework was used to determine the eligibility of the primary research question as shown on Table 1.

**Table 1:** Framework for determining eligibility for the research questions

Population	Intervention	Comparison	Outcomes
Patients in low and middle-income countries	Home-based HIV testing and counseling (HTC)	Facility-based HIV testing	• Primary outcome: use of Home-based HTC. • Secondary outcomes: feasibility and effectiveness

### Inclusion criteria

1. Articles reporting on evidence of patients involved in home-based HTC in LMICs
2. Studies reporting on evidence of use of home-based HTC as an intervention
3. Articles reporting on evidence comparing home-based HTC with facility-based HTC

### Exclusion criteria

1. Studies that do not include patients
2. Studies that do not include home-based HTC as an intervention
3. Studies that are conducted in high-income countries

### Study selection

Study selection occurred in three stages. First stage, one reviewer screened the titles from the databases with guidance from the eligibility criteria. Following title screening, two independent reviewers (MPM and NG) undertook screening of abstracts and full articles. Discrepancies in reviewers’ responses at abstract screening stage were resolved through a discussion until consensus was reached. Discrepancies in reviewers’ responses at full article screening stage were resolved by involving a third screener (DK).

### Data extraction

We used a standardized data extraction sheet to extract data from included studies. We extracted data on the following: author and year of publication, population, percentage of women, and percentage of men, age, type of home-based HTC, name of HTC test manufacturer, study design, geographical area, interventions, study setting (rural/urban/semi-urban), country and income levels, and outcomes as shown on Table 2.

**Table 2:** Characteristics and findings of the included studies

Author and Date	Population	Total participants	Percentage(%) of women	Percentage (%) of men	Age	Type of home-based HTC intervention (supervised or unsupervised)	Type of HTC test (name of test and manufacturer)	Study design	Income level	Geographic setting (rural/urban/semi-urban)
Becker et al. 2014 [16]	couples	390	51	49	15 to 49 years.	Supervised HTC by health care worker	Unigold rapid test	Cross-sectional survey	Low income level	Semi-urban
Bogart, 2016 [28]	home-based testing clients identified	9613	46	54	18–40 years	Supervised HTC by health care worker	Rapid test Manufacture not specified	Descriptive qualitative study	low income level	Rural
Doherty T,2013 [17]	Individuals	4154	74.4	25.6	14 years or more	Supervised HTC by health care worker	SD-Bioline & SENSA tri-line rapid test	Cluster randomized controlled trial.	Low income	Rural
Helleringer et al.2013 [18]	Adults	764	54.7	45.3	≥25	Supervised HTC by health care worker	Determine and Unigold rapid test	Cohort study	low income	Can’t tell
Jürgensen et al.2013 [19]	Individual	1694	54	46	≥16	Supervised HTC by health care worker	Unigold rapid test	cluster-randomized trial	Low income	Rural
Kim,2016 [24]	1850 individuals and 1009 households	1850	Not specified	Not specified	Adult male (aged 20 to 59), an adult female (aged 20 to 49), and an adolescent (aged 15 to 19).	Supervised HTC by health care worker	Rapid test Manufacture not specified	Randomized Controlled Trial	low	Rural
Kohler,2014 [20]	Women	405	100	0	14 years and older	Supervised HTC by health care worker	Wilcoxon Mann-Whitney test	Cross-sectional survey	low income	Rural
Krakowiak, 2015 [29]	partners of pregnant women	1101	85	15	24-31 years	Supervised HTC by health care worker	Unigold KHB rapid test	Randomized clinical trial (with Mix methods)	Low income level	Can’t tell
Krakowiak et al.,2016 [30]	Pregnant women attending their first antenatal. Male partners of women who were.	1101	48	52	24.9 years (SD = 4.9) for women and 31.0 years (SD = 6.4) for men	Supervised HTC by health care worker	Not specified	Randomized clinical trial	low income	Can’t tell
C. Low et al.2013 [27]	Community leaders and Individuals	313	97.1	2.9	average age was 45 years,	Supervised HTC by health care worker	Not specified	cluster-randomized trial	low	Rural
Magasana et al.2016 [31]	individuals	6757	71.9	28.1	14 and above	Supervised HTC by health care worker	Not specified	Randomized control trial	low income	Rural
Ndege et al.2016 [21]	All women aged 13–50 years were considered to be of reproductive age and were included in the analysis.	119.678	100	0	all consenting persons 13 years and older as well as children less than 13 years of age whose mother is either dead, HIV-positive, or whose vital and/or HIV status is of unknown.	Supervised HTC by health care worker	Determine and Unigold rapid test	observational study: Cross-sectional survey	LOW	Rural
Novitsky V et al.2015 [32]	residents aged 16 to 64 for HIV in the north-eastern sector of Mochudi, a community in Botswana with about 44,000 inhabitants	6238	68	32	age range of 16 to 64 years old	Supervised HTC by health care worker	Determine and Unigold rapid test	cohort study	LOW	Semi -urban
Parker et al.2015 [25]	Individuals	12,269	51	49	18 months and above	Supervised HTC by health care worker	Determine and Unigold rapid test	Cross-sectional survey	lower-middle	Rural
Shahid et al.2016 [23]	spouses of HIV-positive men who inject drugs in Pakistan	2400	100	0	17–60	Supervised HTC by health care worker	Determine and Unigold rapid test	Cross-sectional study	Low	Can’t tell
Van Rooyen et al.2016 [26]	All family members (adults, adolescents, and children)	Not indicated	Not indicated	Not indicated	All ages	Supervised HTC by health care worker	Not specified	systematic review	low	Rural
Ruzagira et al.2017 [22]	Five databases were searched for studies published between 1st January 2000 and 19th August 2016 that reported on linkage to care among adults newly identified with HIV infection through home-based HCT	Not indicated	Not indicated	19 articles	Not indicated	Supervised HTC by health care worker	Not specified	systematic review	low income	Can’t tell

### Quality assessment

We utilized the 2011 version of the Mixed Methods Appraisal Tool (MMAT) to assess the risk of bias for the included studies. We assessed the included studies in the following areas: clearness of the research objective or question; ability of collected data to address the research questions; collection of data from suitable sources; and appropriateness and rigor of the statistical analysis. We further assessed the included studies on the thoroughness of assessment of exposures; the researcher’s acknowledgement of potential biases; accuracy of the sampling methodology; representativeness of the population; outcome measurements and conclusion; and the response rate. Research method specific quality assessment was also performed with guidance from the MMAT. Two reviewers (MPM and DK) independently performed the quality assessment. Differences in rating results were resolved by discussion until consensus was reached. An overall percentage quality score was calculated for included studies and scores were interpreted as ≤50%-low quality, 51–75% -average quality, and 76–100% high-quality.

### Collating, summarizing and reporting the results

The study used thematic analysis to report on the findings from existing literature. The included manuscripts were manually coded. The literature was structured around the following themes derived from our study outcomes:

1. **Usage**-defined as the practice of door to door VCT services that occurs in people’s home, where in pre-test counseling to the entire household, obtain consent from eligible member, test and provide results and post-test counseling.
2. **Feasibility-** The state of home-based HIV testing to be practical, convenient and less costly.
3. **Effectiveness of home-based HTC-** The capability of home-based HIV testing and counseling to be successful in producing desired result.

## Results

### Screening results

In all, 1405 eligible studies were identified from a total of 855,117 studies from our initial search (Additional file 1). Of these 1405 studies, 1095 duplicates were deleted. A total of 255 were also excluded after abstracts screening. Full article screening resulted in an exclusion of an additional 38 articles. Of the 38 excluded studies following full article screening, 1 study was conducted in a high-income country (33); 5 studies were conduct before 2013 (9, 34–37), 11 Studies were conducted in LMIC’s but did not report on our intervention which was home-based HTC (38–48) and 21 studies reported no evidence of primary outcome of this study (use of home-based HTC) (7, 10, 34, 49–65).

**Fig. 1:**
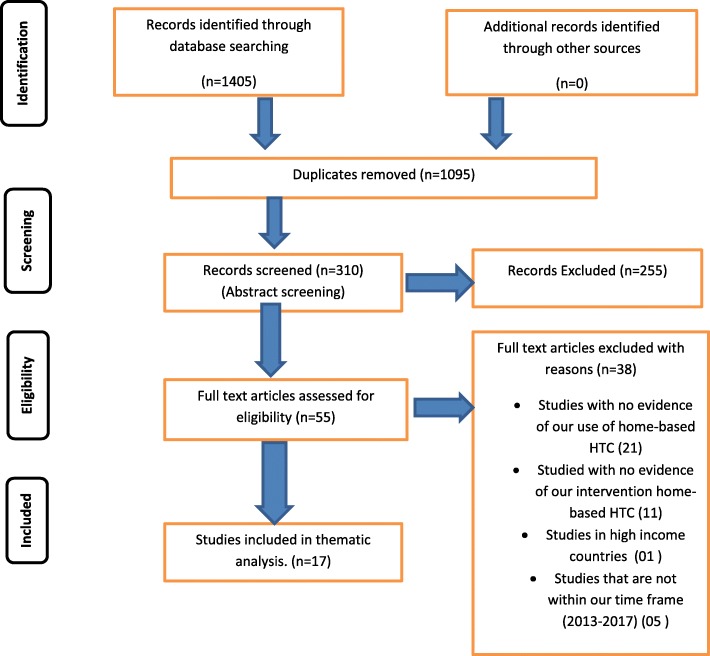
PRISMA flow diagram

Finally, a total of 17 articles were included for data extraction as indicate in Fig. 1. Level of agreement between reviewers was 74.65% versus 71.55% expected by chance which constitutes lack of agreement (Kappa statistic = 0.11, *p*-value> 0.05). However, the McNemar’s chi-square statistic suggests that there was no statistically significant difference in the proportions of yes/no answers by the reviewer (Additional file 2). Discrepancies between reviewers’ responses were resolved by involving a third reviewer.

### Level of bias for included studies

All included studies underwent methodological quality assessment (Additional file 3) using the Mixed Methods Appraisal tool (MMAT)-Version 2011 [30]. All studies scored between 37.5 and 100%. Eight of the 17 included studies scored the highest quality score of 100% [31–38]. Three of the included studies score 50% [39–41]; one study scored the lowest quality score of 37.5% [42] and five of the remaining studies scored between 62.5 and 88.9% [43–47].

### Characteristics of included studies

A total of seventeen studies were eligible for data extraction. Of these, two studies were conducted at a semi-urban setting [31, 48], 10 in rural settings [32, 34–36, 39–42, 46, 47] and five studies did not specify their setting [33, 38, 44, 45, 49]. Of the included studies, three were conducted in south Africa [32, 41, 46], five in Kenya [35, 36, 42, 44, 45], two in Uganda [43, 49], two in Malawi [31, 33], one each in Zambia [34], Botswana [47], Ethiopia [39], Pakistan [38] and Swaziland [40]. All included studies were published between the year 2013 and 2017. The total number of study participants reported in included studies was 9447,025. This number excludes two systematic reviews that did not specify the total number of their population [41, 49]. The age of participants in all included studies ranged from 18 months to 70 years.

The majority (58.6%) of the population included in the study were females. All included studies reported evidence on the use of home-based HTC in LMICs. The included studies used the following study designs: one was a qualitative study [43]; eight were randomized control trials [32, 34, 39, 42, 44–47], five were cross-sectional studies [31, 35, 36, 38, 40]; two were systematic reviews [41, 49], and one cohort studies [33].

### Study findings

#### Usage of home-based HTC

All 17 included studies showed evidence of use of home-based HTC. They all reported on supervised HTC intervention, that is, HTC done by qualified healthcare practitioners. The included studies reported usage of home-based HTC on different HIV tests. A total of 47% of the rapid tests were from Unigold and Determine, 5. 8% were from SD Bio line and SENSA Triline, 5.8% from Wilcoxon Mann-Whitney test, 29% did not specify the type and manufacture and 11.7% used rapid test but did not specify the manufacture. Of the 17 included studies, two study reported on couples home-based HTC [31, 38], one study reported on community-based HTC for Prevention of Mother-to-Child Transmission (PMTCT) [35], one study reported on family-based HTC [41], three studies reported on home-based HTC on pregnant women and their partners [36, 44, 45], five studies reported on the effects of home-based HTC intervention [32, 34, 39, 42, 47]. Of the 17 included studies, seven reported usage of home-based HTC in LMICs [31, 35, 36, 38, 44, 45, 50]. Four studies reported about feasibility of home-based HTC [40, 43, 46, 50]. Lastly, six studies reported on effectiveness of home-based HTC [32–34, 39, 42, 47].

##### Home-based HTC for couples

A study conducted in Malawi reported on couple-home-based HTC [31]. The aim of the study was to estimate the uptake of couple home-based HTC and couple family planning (CFP) services delivered to couples in their homes. A pair of male and female counselors subsequently visited each couple and offered a HIV test [31]. This study reported that 97 (58%) consented to couple home-based HTC, 4 (2%) to couple family planning only and 18 (11%) declined any intervention [31]. Shahid et al., 2016 conducted a study to explore the utility of home and community-based testing of undiagnosed HIV among spouses and children of HIV-positive people who inject drugs [38]. The results showed that, of the 1959 spouses of HIV-positive people who inject drugs, 1896 (96.7%) consented to home and community-based HTC [38]. The study further found that home-based HTC was an effective way of expanding access and identifying cases of undiagnosed HIV among spouses of people who inject drugs (PWID) [38]. Adopting a family centered approach for HIV testing and counseling would help to reinforce health promoting messaged and protective behaviors. However, even though home and community-based HIV testing was reported to be an effective means of accessing children of PWID, majority of spouses who were diagnosed positive were reluctant to consent to testing for their children. This is an alarm that children of PWID might be at risk and need to be tested and referred for HIV treatment and care.

Three studies reported evidence on the use of home-based HTC on pregnant women and their partners [36, 44, 45]. Krakowiak et al., 2015 conducted a study on home-based HTC among male partners of expectant mothers aimed at assessing its effectiveness in Kenya [44]. The study showed evidence that home-based HTC improved substantially the uptake of HIV testing among male partners of expectant mothers, improved HIV status disclosure between couples, and increased sero-discordant partner identification [36, 44]. Krakowiak et al., 2016 conducted another study aimed at comparing the efficacy of planned home-based visit with expectant mother and their male partners to written invitations extended to male partners to accompany their wives for HIV couple counselling and testing at the next antenatal visit [45]. Home-based HTC was conducted by a team of 2 health advisors trained in HIV counseling and testing, 1 male and 1 female [45]. The study results demonstrated the efficacy, acceptability, and practicability of planned home-based visits strategy compared to HIV couple counselling and for expectant mothers and their male partners [45]. It was also reported that twelve women in the home-based HTC strategy and six women who participated in the written invitations strategy experienced physical intimate partner violence but did not attribute this to their participation in the study [45]. The linkage between home-based HTC on pregnant women and their partners and intimate partner violence need to be investigated.

##### Home-based HTC for PMTCT

In 2014, Kohler and colleagues conducted a community-based HTC study in Malawi to assess a community’s perspective on the barriers of accessibility to PMTCT and coverage [35]. Their study findings showed that majority of the study participants who were registered as HIV- positive in the home-based HTC register failed to disclose their HIV-positive status [35]. Their study also reported that very few expectant mothers received a completed course although majority of HIV-positive mothers were place on antiretroviral drugs besides late started of antenatal care by many of the pregnant women [35]. Ndege et al., 2016, study also showed that home-based HTC contributes significantly to reduce the incidence of HIV among newborns and additionally, increases PMTCT coverage [36]. There is a need for more studies on home-based HTC for PMTCT in LMICs to help demonstrate the effectiveness of this intervention on PMTCT in these settings.

##### Home-based HTC for families

Van Rooyen et al., 2016 study conducted with the aim to test all family members for HIV, encourage disclosure and facilitate linkage to care showed that family-based HTC treats the family as a social environment (not just a location for service delivery), through which HIV prevention, treatment, adherence, and support could be achieved [41]. The study further showed that family based HIV testing and counseling increases the identification of HIV-positive children before they become sick and enabling early linkage to care [41]. Despite the increase in the identification of HIV-positive and linkage to care home-based HTC has on the family, nothing has been said on the consequences of this intervention on the family well-being.

#### Feasibility of home-based HTC

Four studies reported the feasibility of home-based HTC [40, 41, 43, 46]. Parker et al., 2015 conducted a study in Swaziland to assess the feasibility and effectiveness of two community-based HTC models in rural Swaziland [40]. The results of this study found mobile-and home-based HTC to be feasible and affordable ways to reach a substantial number of people [40]. Bogart et al. 2016 showed that home-based testing is a feasible and acceptable model for fisher folk communities that can complement existing event-based testing models by encouraging testing among different types of clients [43]. Studies conducted in South Africa, also reported the feasibility of home-based HTC in LMICs [41, 46]. Despite the reported feasibility of the home-based HTC, linkage to care needs to put more emphasis on initiation of anti- retroviral to reduce attrition between testing and treatment initiation.

#### Effectiveness of home-based HTC on patient centered outcomes

Five studies showed evidence on effectiveness of home-based HTC on patient-centered outcomes [32, 34, 39, 42, 47]. Jürgensen et al., (2013) conducted a study to investigate the impact of HIV testing on stigma and to investigate whether home-based HTC has a larger impact on stigma than standard testing services in Malawi [34]. The study observed a reduction in overall reported stigma over time in this rural community. This reduction was most prominent in the items measuring individual attitudes regarding equal rights and respectful treatment for people with HIV/AIDS, that is symbolic stigma [34]. Helleringer et al., (2013) study confirms that home-based HTC campaigns may be one of the most effective strategies to make progress towards universal access targets in Sub-Saharan settings [33].

A study reported that testing rates (supervised VCT) were higher in communities reporting higher stigma, and individuals from high-stigma communities were less likely to have a previous test (Supervised VCT) [42]. It was also demonstrated that although home-based HTC testing increased feelings of anger among HIV-positive individuals, it lowered the sense that having HIV was a sign of immoral behavior [42]. In rural South Africa, Doherty et al., (2013) conducted a study to assess the effect of home-based HTC on the prevalence of HIV testing and reported behavioral changes [32]. This study reveal a moderately high levels of stigma, with over a third of control participants reporting that people with HIV are treated badly owing to their status and almost half observed stigmatizing behavior towards someone with HIV/AIDS [32]. Novitsky et al., 2015 study also reported a lower HIV prevalence rate, 10.9% (95% CI 9.5–12.5%) among individuals tested for the first time using home-based HIV testing in a peri-urban community in Botswana.

Kim et al., 2014 conducted a study in Ethiopia with the aim of studying the causal effects of HIV education, home-based voluntary counseling and testing, and conditional cash transfer for facility-based VCT on HIV/AIDS knowledge and demand for HIV testing [39]. The results showed home-based HTC increases test uptake to a limited extent [39]. However, when HIV/AIDS education is combined with either home-based VCT or CCT for facility-based VCT, testing uptake increased substantially by about 63 and 57 percentage points, respectively [39]. The effectiveness of combining HIV/AIDS education and VCT simultaneously need to be examined.

## Discussion

This scoping review mapped available literature on the use of home-based HTC in LMICs and provided a general overview of research evidence on usage of home-based HTC. Evidence show the use of the following home-based HTC strategies: couple home-based HIV testing and counselling; community based HIV testing and counselling in LMICs. It also provides evidence on feasibility of home-based HTC and effectiveness of home-based HTC in LMICs. The findings of this study show high levels of evidence on the use of home-based HTC by females and by people in rural setting of LMICs. This is probably because the population of women is higher than men in the world [51]. HIV prevalence is mostly high in rural areas because they have limited resources. South Africa and Kenya showed a high level of research evidence of the usage of home-based HTC as compared to other LMICs. This may be because South Africa has reported to have a higher HIV prevalence of 12.2% increases over the 2008 estimate [52]. UNAIDS estimated that 38.6 million people were living with HIV during 2006, with 4.1 million new infections in Kenya [53]. HIV prevalence has recently fallen in some previously affected LMICs, including Uganda [54]. However, no studies reported on the use of home-based HTC in urban settings and only two studies reported evidence of use of home-based HTC in semi-urban setting [31, 47]. This demonstrates a gap in literature regarding the use of home-based HTC in urban and semi-urban settings.

Recent WHO’s guidelines embolden greater public health importance on couple counselling and testing [55]. Sero-discordant transmission of HIV between couples contributes significantly to new HIV morbidities in Sub-Saharan Africa [1]. In view of this, shared knowledge of ones HIV status among couples is highly recommended by the WHO guidelines on couple counselling and testing in order to reduce HIV new infections among sero-discordant couples [56]. The findings of our study show five studies on the use of home-based HTC for couples as well as the acceptability of this strategy. Home –based HTC is has been demonstrated to be the most accepted and preferred by expectant mothers and their partners compared to others such as facility-based and VCT-center based HTC of partners [57]. However, our findings show that home-based HTC for couples poses a challenge such as domestic violence for couples who test positive. The provision of home-based VCT should therefore be complemented with an HIV risk assessment particularly for particularly for couples with sexually transmitted infections and couples encouraged to subsequently seek appropriate care at a health facility [58].

This study shows that home-based HTC is accessible to a substantial number of people and have the potential to increase the number of people who know their HIV status in rural and low-resourced HIV hyper-pandemic settings [40]. However, it has been demonstrated that there are fewer number of men who use home-based HTC in comparison to women. The WHO recommends the implementation of a combination of facility- and community-based models of home-based HTC in generalized epidemics to reach all populations including men [13]. Although Mulogo et al., (2011) study indicated that the provision of VCT in a home setting instead of facility-based testing is likely to lead to failure or a delay in identification of illnesses that may be risk factors for HIV, it further suggested that potential clients should be provided with both facility and home based VCT service options within a given setting [58].

### Implications for practice

Majority of the included studies were conducted in a rural setting where access to healthcare and laboratory infrastructure is either not available or poorly developed coupled with lack/inadequate skilled healthcare professional [32, 34–36, 39–42, 46, 48, 43, 59–62]. Bearing in mind the poor accessibility of primary healthcare facilities and laboratory infrastructure as well as the high prevalence of HIV in the LMICs, increasing the promotion of home-based HTC in both is recommended in these settings. This would help improve accessibility of HIV testing and increase the number of people who know their HIV status and enable LMICs with achieving the UNAID 90:90:90 goal which aims at achieving, 90% of all people living with HIV to know their HIV status, 90% of all people with diagnosed HIV infection to be receiving sustained antiretroviral therapy and 90% of all people receiving antiretroviral therapy to have a viral suppression by the year 2020 [63].

### Implications for research

Our study shows that there is limited published research specific to the usage of home-based HTC in urban and semi-urban settings, indicating a need for more research on the use of home-based HTC in these settings. These studies would provide a contextual insight on the use of home-based HTC in urban and semi-urban setting. This would also help reveal facilitator, barriers and challenges related to use of home-based HTC in LMICs urban settings. Our study also revealed that there is a need for studies of usage of home-based HTC on men. This would improve the accessibility of HIV testing and counseling for hard-to-reach populations such as men.

### Strength and limitations

This study included studies conducted in different settings (rural, urban, and semi-urban), which gives a picture of the operational experiences and challenges that could be encountered when home-based HTC is undertaken in similar settings. The scoping review methodology allowed for the inclusion of all types of study designs and used a systematic approach to identifying relevant studies, charting, and for analyzing the outcomes [25] which may not be done in other types of reviews such as expert reviews and literature reviews. Another important strength of the study is the exhaustive search for relevant studies without limiting the language which is mostly the case in primary studies. This study was however limited in that, we only included studies that were conducted between the year 2013 and 2017 and excluded studies conducted before 2013.

## Conclusions

This study demonstrates that home-based HTC plays a vital role in facilitating the uptake of HIV testing in LMICs, particularly in HIV hyper-endemic settings. This study in addition shows that there is a need for more research on the use of home-based HTC for males and for urban and semi-urban settings hence, further studies are recommended.

## Additional files

Additional file 1: Electronic Search results for title screening. (DOCX 15 kb)

Additional file 2: Reviewers’ responses at full screening stage and degree of agreement. (DOCX 17 kb)

Additional file 3: Methodological quality assessment. (XLSX 12 kb)

## Abbreviations

HIVST: HIV self-testing
HTC: HIV testing and counseling
LMICs: Low and middle income countries
PMTCT: Prevention of mother-to-child transmission
POC: Point-of-care
VCT: Voluntary counseling and testing
