# Supplementary material for: The use of home-based HIV testing and counseling in low-and-middle income countries: a scoping review
Source: BMC Public Health. 2019 Jan 31;19:132. doi: 10.1186/s12889-019-6471-4 (PMC6357437; doi:10.1186/s12889-019-6471-4)
Supplement: Supplementary file 1 — Electronic Search results for title screening. (DOCX 15 kb) [file 12889_2019_6471_MOESM1_ESM.docx]

**Electronic Search results for title screening**

| **Date of search** | **Key-Words** | **Page** | **Results** | **Included** |
| --- | --- | --- | --- | --- |
| 1**.** 17.03.2017 | Home-based HIV testing and counseling  Acceptability  Use  Feasibility | Ebscohost | 10045 | 25 |
| 2. 24.03.2017 | Home-based HIV testing  Use and acceptability | Google scholar | 828109 | 34 |
| 3. 05.04.2017 | Home-based HIV testing | Pubmed | 86 | 51 |
| 4. 06.04.2017 | Home based HIV testing | Google scholar | 727 | 380 |
| 5.24.04.2017 | Home based HIV testing, patients  And use or acceptability | Ebscohost | 45 | 450 |
| 6. 24.04.2017 | Home based HIV testing, patients  and use and acceptability or  feasibility | Ebscohost | 8912 | 65 |
| 7. 24.04.2017 | Home-based HIV testing and  counseling | WHO | 577 | 30 |
| 8. 28.04.2017 | Home based HIV testing | Science direct | 00 | 00 |
| 9. 30.04.2017 | Home based HIV testing | UNAIDS database | 5630 | 350 |
| 10. 01.05.2017 | Home based HIV testing | WHO | 986 | 20 |
| **Total** |  |  | 855117 | **1405** |
