# Supplementary material for: The use of home-based HIV testing and counseling in low-and-middle income countries: a scoping review
Source: BMC Public Health. 2019 Jan 31;19:132. doi: 10.1186/s12889-019-6471-4 (PMC6357437; doi:10.1186/s12889-019-6471-4)
Supplement: Supplementary file 2 — Reviewers’ responses at full screening stage and degree of agreement. (DOCX 17 kb) [file 12889_2019_6471_MOESM2_ESM.docx]

List of studies included for full article screening and reviewers’ responses

| **Author and Date** | **Response: Reviewer 1 PS** | **Response: Reviewer 2 ND** |
| --- | --- | --- |
| Hanani Tabana 2013 | No | Yes |
| Mosa Moshabela 2016 | No | Yes |
| Laura M. Bogart 2016 | Yes | Yes |
| Tabana,2015 | Yes | Yes |
| C. B. Deery 2014 | Yes | No |
| Chamie, 2016 | Yes | Yes |
| Hayes,2017 | No | Yes |
| Lippman,2014 | Yes | Yes |
| Plazy,2016 | Yes | Yes |
| Magasana,2016 | Yes | Yes |
| Ruzagira,2017 | Yes | Yes |
| Shumba,2013 | Yes | Yes |
| Eaton,2016 | No | No |
| Kenya,,2016 | No | No |
| Naik,2012 | Yes | Yes |
| Gliemann,2016 | No | Yes |
| Kohler,2014 | Yes | Yes |
| Chang,2016 | No | Yes |
| Mulogo,2013 | Yes | Yes |
| Musekwa,2014 | No | Yes |
| Kalumbi1,2014 | Yes | Yes |
| McGovern,2016 | Yes | Yes |
| Medley,2012 | Yes | Yes |
| Doherty,2013 | Yes | Yes |
| Krakowiak,2015 | Yes | Yes |
| Jürgensen,2013 | Yes | Yes |
| Knight,2016 | Yes | Yes |
| Novitsk.2015 | Yes | Yes |
| Drain, 2015 | No | Yes |
| Shahid ,2016 | No | Yes |
| Mulogo,2011 | yes | Yes |
| Maman,2016 | Yes | Yes |
| Hensen,2015 | No | Yes |
| Pettifor,2015 | Yes | No |
| Parker, 2015 | Yes | Yes |
| van Rooyen,2014 | Yes | Yes |
| Ndege,2016 | Yes | Yes |
| Wachira,,2014 | Yes | Yes |
| Driver,2017 | Yes | Yes |
| Chimoy,2015 | Yes | No |
| Osoti,2015 | Yes | Yes |
| Kyaddondo,2012 | Yes | Yes |
| Krakowiak, 2016 | Yes | Yes |
| Alfred Onyango Osoti 2015 | Yes | Yes |
| Tao,2014 | yes | Yes |
| Labhardt,2014 | yes | Yes |
| Aliyu,2016 | No | No |
| Naik,2015 | Yes | Yes |
| Ruzagira,2017 | Yes | Yes |
| Ferrand,2015 | Yes | Yes |
| Sharma,2016 | Yes | Yes |
| van Heerden,2017 | Yes | Yes |
| Becker,2014 | Yes | No |
| Duong, 2014 | No | Yes |
| Kim,2016 | Yes | Yes |
| Jackson, 2013 | Yes | Yes |
| Helleringer,2014 | Yes | Yes |
| MacPherson, 2011 | Yes | Yes |
| Kenya,2016 | Yes | No |
| Fylkesnes,2013 | Yes | Yes |
| Justman,2017 | No | Yes |
| van Rooyen,2016 | Yes | Yes |
| Low,2013 | Yes | Yes |
| Bigogo,2014 | Yes | No |
| Jürgensen,2013 | Yes | Yes |
| Ng’ang’a,2014 | Yes | Yes |
| Mantell,2014 | Yes | Yes |
| Witzel,2017 | Yes | Yes |
| Iwuji, 2016 | Yes | Yes |
| Choko,2015 | No | Yes |
| Shanaube,2017 | Yes | Yes |

kap ResponseReviewer1PS ResponseReviewer2ND

Expected

Agreement Agreement Kappa Std. Err. Z Prob>Z

-----------------------------------------------------------------

74.65% 71.55% 0.1088 0.1133 0.96 0.1685

. mcc ResponseReviewer1PS ResponseReviewer2ND

| Controls |

Cases | Exposed Unexposed | Total

-----------------+------------------------+------------

Exposed | 50 6 | 56

Unexposed | 12 3 | 15

-----------------+------------------------+------------

Total | 62 9 | 71

McNemar's chi2(1) = 2.00 Prob > chi2 = 0.1573

Exact McNemar significance probability = 0.2379

Proportion with factor

Cases .7887324

Controls .8732394 [95% Conf. Interval]

--------- --------------------

difference -.084507 -.2140488 .0450348

ratio .9032258 .7843463 1.040123

rel. diff. -.6666667 -1.859463 .5261294

odds ratio .5 .1539714 1.439469 (exact)

**Interpretation of your results (You will need to put the statement below in the results section).**

74.65% agreement versus 71.55% expected by chance which constitutes lack of agreement (Kappa statistic = 0.11, p-value=0.17). However, the McNemar's chi-square statistic suggests that there is not a statistically significant difference in the proportions of yes/no answers by reviewer.
